# Patient Satisfaction With a Novel Postcoital Intravaginal Fluid-Absorbing Device

**DOI:** 10.1097/og9.0000000000000190

**Published:** 2026-07-30

**Authors:** Michael Ingber, Anne L. Ackerman, Rachel Rubin, Ryan Sobel

**Affiliations:** Department of Surgery, Atlantic Health System, Morristown, New Jersey; Department of Urology, Rutgers New Jersey Medical School Newark, New Jersey; Department of Urology, UCLA David Geffen School of Medicine, Los Angeles, California; Department of Urology, Georgetown University, Washington, DC; and Jefferson Vulvovaginal Health Center, Department of Obstetrics and Gynecology, Sidney Kimmel Medical College at Thomas Jefferson University, Philadelphia, Pennsylvania.

## Abstract

**OBJECTIVE:**

To evaluate patient-reported symptom outcomes, usability, and vaginal pH after use of a postcoital intravaginal fluid-absorbing device.

**METHODS:**

In this prospective cohort study, adult women who were sexually active with male partners used a postcoital intravaginal fluid-absorbing device after intercourse. Participants completed baseline symptom assessments and a postuse questionnaire evaluating discharge, odor, usability, and likelihood of reuse. A subset performed paired vaginal pH measurements at baseline and at 2–6 and 10–14 hours after intercourse under two conditions: intercourse without the device and intercourse followed by device use. Paired *t* tests were used for comparisons.

**RESULTS:**

A total of 542 participants enrolled; 291 (53.7%) completed the postuse questionnaire. Baseline postcoital symptoms were common, including discharge or dripping (87.6%), vaginal odor (64.9%), and a sensation of feeling unclean (58.1%). Among respondents, 98.0% reported improvement in postcoital discharge and 84.3% reported improvement in vaginal odor after device use. Most participants reported they would use the product again (93.1%) and recommend it to others (94.2%). Usability ratings were favorable, with 88.3% reporting ease of insertion and 93.1% reporting ease of removal. In the paired pH subset (n=48), mean vaginal pH was lower with device use compared with no device at 2–6 hours (5.2±0.9 vs 5.5±0.9, *P*=.025) and at 10–14 hours (4.6±0.6 vs 5.1±0.8, *P*<.001).

**CONCLUSION:**

Use of a postcoital intravaginal fluid-absorbing device was associated with improvement in patient-reported postcoital discharge and odor, high user acceptability, and lower vaginal pH after intercourse. These findings support further evaluation of nonpharmacologic approaches to managing common postcoital gynecologic symptoms.

**CLINICAL TRIAL REGISTRATION:**

ClinicalTrials.gov, NCT06530186.

## Abstract 2

A postcoital intravaginal fluid-absorbing device was associated with improved patient-reported discharge and odor, as well as faster normalization of vaginal pH after intercourse.

Although female sexual health domains such as libido, arousal, and dyspareunia have been extensively studied, formal epidemiologic data regarding postcoital hygiene concerns remain limited. Prior observational work and patient-reported concerns suggest that postcoital discharge, semen leakage (“dripping”), vaginal odor, and residual fluid discomfort may represent underrecognized quality-of-life issues for some sexually active women.^1,2^ Although these symptoms are not typically associated with a defined pathologic condition, they may be bothersome and negatively affect postcoital comfort and quality of life. Despite their apparent prevalence, these concerns remain underrecognized in the literature, and clinicians do not consistently assess them during standard evaluations.^3^ As a result, patients have limited access to evidence-based, nonpharmacologic strategies for managing postcoital symptoms.

Semen exposure may influence the vaginal environment and may contribute to postcoital symptoms. Semen is alkaline and can transiently increase vaginal pH after intercourse, with potential effects on host defense and the vaginal microbiome.^4,5^ Prior studies have demonstrated associations between semen exposure and changes in vaginal flora, including conditions such as bacterial vaginosis, as well as increased vaginal malodor.^6–8^ Conversely, a *Lactobacillus*-dominant vaginal microbiome is typically associated with lower vaginal pH and is considered a marker of vaginal health.^9^ These observations suggest that the duration of semen retention within the vaginal canal may influence postcoital symptoms, although this relationship has not been well studied in the context of symptom-directed interventions.

Despite the frequency of postcoital symptoms, few nonpharmacologic approaches have been evaluated specifically for their management. Many available products marketed for vaginal or sexual health aim to modify vaginal pH or support the vaginal microbiome but do not directly address the presence of semen and other fluids within the vaginal canal after intercourse. A postcoital intravaginal fluid-absorbing device was developed to remove semen and associated fluids after intercourse. The device consists of a tampon-like applicator and a polyurethane foam element designed to expand within the vaginal fornices, where fluid may be retained. The objective of this prospective cohort study was to evaluate patient-reported symptom outcomes, usability, and vaginal pH following use of this device after intercourse. We hypothesized that device use would be associated with improvement in postcoital discharge and odor, high user acceptability, and a more rapid return of vaginal pH toward baseline compared with intercourse without device use.

## METHODS

**Fig. 1.**
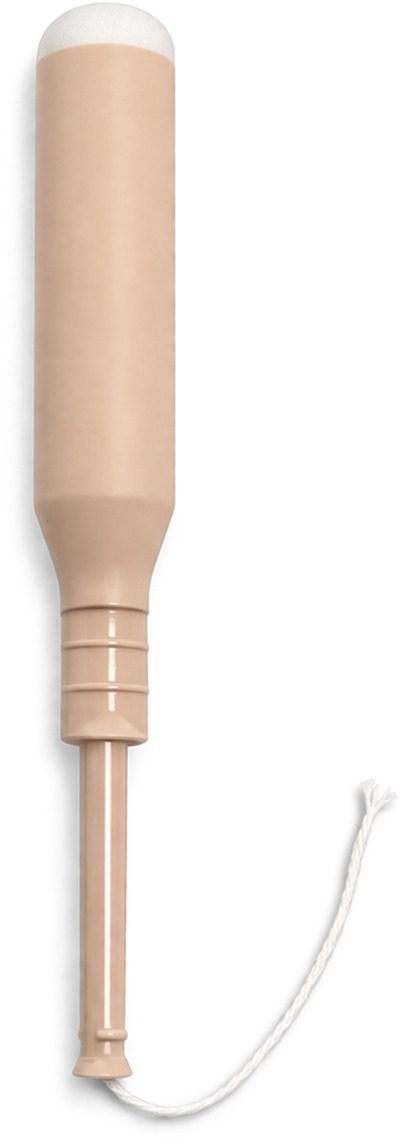
Intravaginal fluid-absorbing device in its predeployment state, consisting of a polyethylene applicator, plunger, and absorbent polyurethane foam tip before expansion. In contrast to a traditional cotton or rayon tampon, the device incorporates an open-tip applicator design, a longer applicator profile, and an absorbent polyurethane foam component intended for postcoital fluid absorption rather than menstrual fluid collection. Image courtesy of LiviWell, Inc. Used with permission.

**Fig. 2.**
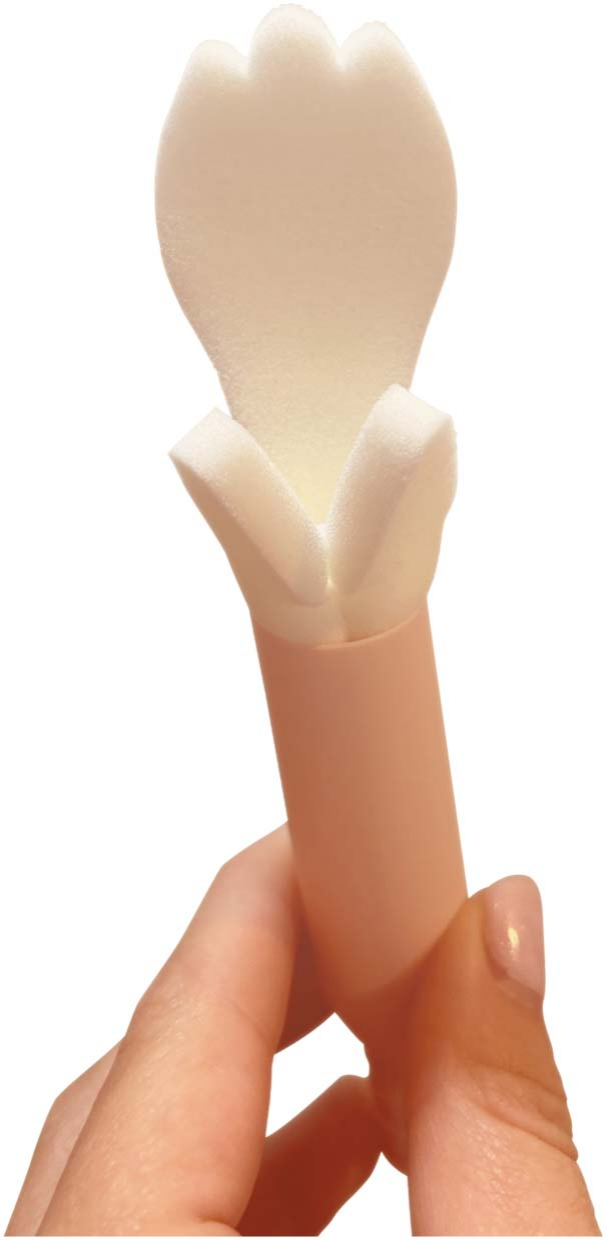
Deployed configuration of the device. Expanded polyurethane foam tip demonstrating the tulip-shaped configuration designed to conform to the vaginal fornices for fluid absorption after insertion. Image courtesy of LiviWell, Inc. Used with permission.

We performed a prospective cohort study using an IRB-approved clinical protocol. The study was approved by the Salus IRB. Participants reviewed an electronic informed consent document and provided informed consent through a Health Insurance Portability and Accountability Act–compliant electronic platform (Jotform) before enrollment. A signed copy of the consent form was automatically emailed to participants for their records. The investigational device evaluated in this study (Livi) is an intravaginal absorbent device designed for postcoital fluid absorption. Although similar in concept to a tampon, the device is slightly longer and is intended to reach the vaginal fornices. It consists of a soft, rapidly absorbing polyurethane foam rather than the tightly woven cotton or rayon fibers used in conventional tampons (Fig. 1). On deployment, the foam expands into a configuration designed to conform to the vaginal fornices (Fig. 2). At the time of manuscript submission, the device had received clearance from the U.S. Food and Drug Administration through the 510(k) pathway for the absorption of semen and vaginal discharge.

*Ingber. Postcoital Fluid-Absorbing Device Outcomes. O&G Open 2026*.

*Ingber. Postcoital Fluid-Absorbing Device Outcomes. O&G Open 2026*.

Adult women 18 years of age or older were eligible if they were sexually active with male partners, willing and able to use the study product, resided in the continental United States, and could read and write English. Exclusion criteria included pregnancy or attempts to conceive, use of condoms during intercourse, inability to use the device because of poor motor function, and inability to provide informed consent. The protocol characterized the device as a nonsignificant-risk device and described the product as a tampon-like synthetic absorbent intended to absorb semen and other vaginal fluids after intercourse. This study included a nonrandomized, within-subject comparison in a subset of participants who completed pH measurements under both device and no-device conditions.

Participants were recruited through clinic-based and online outreach. For consenting patients, study devices were mailed directly to participants. Participants were not compensated for the primary mailed survey, and the intervention and outcome collection was performed remotely. Participants completed baseline demographic and symptom questionnaires. Demographic variables, including age, race and ethnicity, education, income, and sexual frequency, were collected to characterize the study population and to assess the generalizability of the findings. Participants were also asked whether they had previously experienced postcoital discharge/dripping, vaginal odor, a sensation of feeling unclean, urinary tract infection, bacterial vaginosis, or yeast infection after sexual activity. Subjects were instructed to insert the device as they would a tampon as soon as possible after ejaculation. In this study, participants were instructed to use the device for a minimum of 1 minute and a maximum of up to 1 hour after intercourse. Subsequent to study completion, labeling for the commercially available device was finalized with a maximum recommended dwell time of 15 minutes. Participants were then asked to scan a QR code and to complete an electronic survey the following day after use.

A subset of study participants 18–45 years of age who were willing to check vaginal pH at home at two postcoital time intervals were offered $25 in the form of an Amazon gift card for their time. In the pH subset, participants underwent baseline vaginal pH assessment in the office setting before home-based postcoital testing. Participants were provided standardized written instructions describing vaginal pH collection technique, timing intervals, and data reporting procedures. Participants then performed self-measured vaginal pH assessments approximately 2–6 and 10–14 hours after intercourse under two conditions: intercourse without the device and intercourse followed by device use. After completing measurements, participants recorded pH values electronically using a QR code–linked study survey. Vaginal pH was self-measured with commercially available pH test strips (Vaginox), which provide colorimetric readings across a physiologic vaginal pH range (approximately 3.8–7.0) in discrete increments. Participants were instructed to compare the strip color to the provided reference scale immediately after sampling and to record the corresponding pH value. Although the order of device and no-device conditions was not randomized, participants were instructed to have a 48-hour interval between intercourse with and without the study device to minimize carryover effects. The principal comparisons for this study were no-device versus device pH at 2–6 and 10–14 hours after intercourse.

Descriptive statistics were used to summarize demographic characteristics, baseline symptoms, and patient experience questionnaire responses. Categorical variables were presented as counts and percentages. For the vaginal pH subset, paired analyses were performed comparing measurements obtained after intercourse with and without device use at corresponding time points. Mean pH values were reported as mean±SD. Paired two-tailed *t* tests were used to compare pH values between conditions at 2–6 and 10–14 hours after intercourse. In the pH subset, mean change from baseline vaginal pH was also calculated for each postcoital time point under both study conditions. Paired *t* tests were used to compare change from baseline values between conditions. Mean paired differences and corresponding 95% CIs were calculated.

The protocol originally targeted enrollment of approximately 100 participants in the pH subset to provide 80% power to detect a 0.3-unit difference in pH between conditions. This effect size was selected because even modest changes in vaginal pH may influence the vaginal microenvironment and microbial composition after intercourse. However, completion of all required pH measurements was voluntary and performed at home, resulting in incomplete follow-up for some participants. Analyses were therefore performed on participants with complete paired measurements (n=48).

All statistical analyses were performed with Microsoft Excel for paired comparisons with a significance threshold of *P*<.05.

## RESULTS

A total of 542 women were enrolled in the study. Of these, 291 participants (53.7%) completed the postuse questionnaire and made up the analytic cohort. Baseline demographic and sexual behavior characteristics are summarized in Table 1. Most participants were under 55 years of age (26–40 years of age 45.0%, 41–55 years of age 41.9%), and the majority identified as White (77.7%). The most reported frequency of sexual activity was multiple times per week (45.7%), followed by weekly (35.4%).

**Table 1.** Baseline Demographic Characteristics of the Analytic Cohort (n=291)

	n (%)
Age (y)	
18–25	13 (4.5)
26–40	131 (45)
41–55	122 (41.9)
Over 55	25 (8.6)
Race and ethnicity	
Asian/Pacific Islander	3 (1)
Black or African American	24 (8.2)
Hispanic or Latino	25 (8.6)
White	226 (77.7)
Additional races and ethnicities/did not answer	13
Income ($)	
More than 150,000	55 (18.9)
50,000–150,000	114 (39.2)
Less than 49,999	101 (34.7)
Prefer not to answer	21 (7.2)
Education	
What is the highest degree or level of school you have completed? (If currently enrolled, highest degree received)	
Graduate degree or higher	40 (13.7)
Bachelor's degree	58 (19.9)
Some college	103 (35.4)
High school or equivalent	84 (28.9)
Other	6 (2.1)
Sexual frequency	
On average, how frequently do you have sexual intercourse?	
Daily	26 (8.9)
Multiple times per week (2–5 times/wk)	133 (45.7)
Weekly (once per week)	103 (35.4)
Monthly (once per month)	23 (7.9)
A few times per year (every few months)	6 (2.1)

Baseline postcoital symptoms were frequently reported (Table 2). A history of postcoital discharge or dripping was reported by 87.6% of respondents; 64.9% reported vaginal odor; and 58.1% reported a sensation of feeling unclean after intercourse.

**Table 2.** Baseline Postcoital Complaints Reported by the Analytic Cohort (n=291)

Symptoms	n (%)
After sexual activity, which of the following have you experienced in the past?	
Discharge or dripping of sexual fluids (semen, lubricant)	255 (87.6)
Vaginal odor	189 (64.9)
Sensation of feeling unclean	169 (58.1)
Urinary tract infections	108 (37.1)
Bacterial vaginosis	68 (23.4)
Yeast infections	61 (21)

Ease-of-use ratings for the device were favorable (Table 3). Most participants agreed or strongly agreed that the device was easy to insert (88.3%) and easy to remove (93.1%), that the packaging was easy to open (95.9%), and that the instructions were easy to understand (96.6%). Most respondents also reported that they would use the product again (93.1%) and would recommend it to a friend (94.2%).

**Table 3.** Usability Questions

	Strongly Agree	Agree	Neither Agree nor Disagree	Disagree	Strongly Disagree
The product was easy to insert	172 (59.1)	85 (29.2)	11 (3.8)	13 (4.5)	10 (3.4)
The product was easy to remove	188 (64.6)	82 (28.2)	5 (1.7)	7 (2.4)	8 (2.7)
The packaging was easy to open	208 (71.5)	71 (24.4)	6 (2.1)	1 (0.3)	5 (1.7)
The instructions were easy to understand	213 (73.2)	68 (23.4)	5 (1.7)	2 (0.7)	3 (1.0)

	Very Likely	Somewhat Likely	Neither Likely nor Unlikely	Somewhat Unlikely	Very Unlikely
How likely would you be to use this product again?	204 (70.1)	67 (23.0)	11 (3.8)	5 (1.7)	4 (1.4)
How likely would you be to recommend this product to a friend?	199 (68.4)	75 (25.8)	10 (3.4)	4 (1.4)	3 (1.0)

Data are presented as n (%).

Among participants who completed the symptom outcome items, 98.0% reported at least some improvement in postcoital dripping or discharge after device use (Table 4). Nearly half of respondents (47.6%) reported that symptoms were very much better, with an additional substantial proportion (39.6%) reporting that they were much better. Improvement in vaginal odor was also commonly reported, with 84.3% of respondents indicating at least minimal improvement. In addition, 63.2% of respondents reported feeling less worried or anxious about sex after using the device, and 68.3% indicated that their frequency of sexual activity might increase if the product were regularly available (Table 4).

**Table 4.** Patient-Reported Outcomes and Usability After Product Use

	Very Much Better	Much Better	Minimally Better	No Change	Minimally Worse	Much Worse	Very Much Worse
After using the product, how would you describe your vaginal discharge after sex (subjects with baseline discharge only)?	119 (47.6)	99 (39.6)	27 (10.8)	4 (1.6)	1 (0.4)	0 (0)	0 (0)
After using the product, how would you describe your vaginal odor after sex (subjects with baseline odor only)?	90 (33.6)	102 (38.1)	34 (12.7)	42 (15.7)	0 (0)	0 (0)	0 (0)

	Strongly Agree	Somewhat Agree	Neither Agree nor Disagree	Somewhat Disagree	Strongly Disagree
After using the product, I felt less worried and anxious about sex.	88 (30.6)	94 (32.6)	83 (28.8)	13 (4.5)	10 (3.5)
If this product were regularly available, I would expect my frequency of sex to increase.	100 (34.5)	98 (33.8)	71 (24.5)	11 (3.8)	10 (3.4)

Data are presented as n (%).

Forty-eight subjects completed baseline and all postcoital vaginal pH measurements and were analyzed (Table 5). Baseline demographic characteristics of the pH subset were similar to those of the larger analytic cohort, including age distribution, race and ethnicity, and reported sexual frequency. Mean baseline vaginal pH was 4.4±0.7. A single baseline vaginal pH measurement was obtained before postcoital testing and was used as the reference value for calculation of change from baseline outcomes under both study conditions. Mean vaginal pH was lower with device use compared with no device use at 2–6 hours (5.2±0.9 vs 5.5±0.9, *P*=.025) and at 10–14 hours (4.6±0.6 vs 5.1±0.8, *P*<.001). Compared with baseline vaginal pH, intercourse without device use resulted in a greater postcoital elevation in vaginal pH than intercourse with device use. Mean change from baseline pH at 2–6 hours was +1.03 without device use versus +0.74 with device use (*P*=.022). At 10–14 hours, mean change from baseline pH was +0.64 without device use versus +0.14 with device use (*P*<.001). The mean paired difference in change from baseline was 0.29 pH units (95% CI, 0.04–0.53) at 2–6 hours and 0.50 pH units (95% CI, 0.31–0.70) at 10–14 hours.

**Table 5.** Paired Vaginal pH Outcomes in Complete pH Datasets (n=48)

Time Point	Without Device*	With Device*	Δ From Baseline Without Device	Δ From Baseline With Device	*P*
Baseline	4.4±0.7	—			—
2–6 h	5.5±0.9	5.2±0.9	+1.03	+0.74	.025
10–14 h	5.1±0.8	4.6±0.6	+0.64	+0.14	<.001

*P* values represent paired comparisons of vaginal pH between device and no-device conditions at the corresponding postcoital time point.

* Data are presented as mean±SD.

## DISCUSSION

In this prospective remote cohort study, use of a novel tampon-like postcoital fluid-absorbing intravaginal device was associated with high rates of self-reported improvement in two common postcoital gynecologic symptoms: persistent vaginal discharge and vaginal odor. Usability metrics were also favorable, with most respondents indicating that the novel product was easy to insert, easy to remove, and understandable with the supplied instructions.

Vaginal pH increased after intercourse in all groups, consistent with prior studies showing that semen transiently alkalinizes the vaginal environment, which can impair components of innate host defense.^5^ In this study, device use was associated with lower pH both at 2–6 and 10–14 hours after intercourse, representing, to the best of our knowledge, among the first clinical evaluations of a device intended to reduce postcoital vaginal alkalinization. pH is not a direct surrogate for the vaginal microbiome or infection risk; however, these findings are consistent with our hypothesis that more rapid semen removal reduces the duration of postcoital vaginal alkalinization. Microbiome analyses were not performed, although rapid restoration of vaginal acidity after intercourse may be consistent with conditions that favor a *Lactobacillus*-dominant vaginal environment. It is important to note that although use of the device was associated with attenuation of postcoital vaginal pH elevation, the clinical implications of these transient physiologic changes remain uncertain and warrant further investigation. These data should not be overinterpreted as proof that the device prevents bacterial vaginosis, urinary tract infection, or other genital tract conditions. However, the results are clinically interesting because the vaginal microbiome and vaginal pH are closely linked, and semen exposure has been associated with microbiologic and immunologic changes that plausibly contribute to postcoital dysbiosis and the development of symptoms. The present study therefore provides a foundation for future controlled investigations incorporating formal microbiome sampling, Nugent scoring, amine profiling, or validated symptom instruments.

Our study highlights an underrecognized clinical issue: Many women report bothersome postcoital symptoms such as persistent discharge, vaginal odor, or a sensation of feeling unclean after intercourse. These concerns are not routinely assessed during clinical encounters and currently lack dedicated nonpharmacologic management strategies. A postcoital intravaginal absorbent device may therefore address a meaningful quality-of-life need for patients experiencing these symptoms.

This study has several notable strengths. It includes one of the largest cohorts to date examining postcoital symptoms in sexually active women. The prospective design allowed standardized collection of baseline symptoms and postuse patient-reported outcomes. In addition, the inclusion of paired physiologic measurements of vaginal pH after intercourse provides objective biologic data supporting the patient-reported findings. The pragmatic design also allowed the device to be evaluated under real-world conditions among participants throughout the United States. For a symptom domain that has received little formal study, these data provide a reasonable foundation for subsequent trials.

This study has several important limitations. Participants were self-selected individuals interested in use of a postcoital fluid-absorption device, which may introduce selection bias and limit generalizability to broader populations. Baseline histories of urinary tract infection, bacterial vaginosis, and yeast infection were self-reported and were not independently verified, introducing the potential for recall bias or diagnostic misclassification. Study completion and postuse survey response rates were incomplete, which may introduce response bias and limit interpretation of participant-reported outcomes. Next, the study protocol allowed device use of up to 1 hour, whereas current labeling recommends a maximum dwell time of 15 minutes. Device dwell time in this study varied among participants, reflecting real-world home-use conditions, and may have contributed to the variability in participant-reported outcomes. This difference reflects poststudy refinements to product labeling and safety-related risk mitigation considerations, including minimization of prolonged intravaginal dwell time and theoretical risks such as irritation, hypersensitivity, or toxic shock syndrome associated with extended device retention. Given that the device is designed for rapid absorption of intravaginal fluid, typically occurring shortly after placement, we believe that this difference is unlikely to materially affect the observed symptom outcomes. The study was a mailed, uncompensated pragmatic cohort study with substantial attrition from enrollment to completed postuse questionnaire (291 responses among 542 enrolled participants), which introduces clear response bias risk. In addition, patient-reported outcome measures used here were study specific because no standard exists for measuring subjective postcoital discharge or odor. The study was not randomized, blinded, or sham controlled. The pH subset was modest in size and relied on self-testing. Broader home collection windows for postcoital pH measurements may have introduced variability in measured vaginal pH values. However, collection intervals were predefined and based on prior published methodology, and paired within-subject analyses still demonstrated statistically significant differences between conditions at both evaluated time points. The study did not systematically collect information on lubricant use, spermicides, or other intravaginal sexual products that may have influenced postcoital fluid characteristics, symptom perception, or vaginal pH measurements. Finally, although the device was associated with lower vaginal pH after intercourse, this study did not directly measure vaginal microbiome composition, infection outcomes, or longer-term sexual health outcomes.

In conclusion, use of a postcoital intravaginal fluid-absorbing device was associated with improvement in patient-reported postcoital dripping/discharge and vaginal odor, high user acceptability, and a lower vaginal pH after intercourse. These findings support continued clinical evaluation of nonpharmacologic approaches to managing common postcoital gynecologic symptoms.
